# Neuroprotective Nanomaterials for Central Nervous System Injury: Beyond Drug Delivery Toward Functional Neurorepair

**DOI:** 10.3390/biomedicines14102382

**Published:** 2026-10-19

**Authors:** Martina Colasante, Luana Di Leandro, Michele D’Angelo, Vanessa Castelli, Francesco D’Egidio, Annamaria Cimini, Rodolfo Ippoliti, Mariano Catanesi

**Affiliations:** 1 Department of Life, Health and Environmental Sciences, University of L’Aquila, 67100 L’Aquila, Italy; martina.colasante@guest.univaq.it (M.C.); luana.dileandro@univaq.it (L.D.L.); michele.dangelo@univaq.it (M.D.); vanessa.castelli@univaq.it (V.C.); francesco.degidio@univaq.it (F.D.); annamaria.cimini@univaq.it (A.C.); rodolfo.ippoliti@univaq.it (R.I.); 2 Sbarro Institute for Cancer Research and Molecular Medicine, Department of Biology, Temple University, Philadelphia, PA 19122, USA

**Keywords:** neuroprotective nanomaterials, functional neurorepair, neuroregeneration, central nervous system injury, nanomedicine

## Abstract

Neurological disorders and central nervous system (CNS) injuries, including neurodegenerative diseases, ischemic stroke, and traumatic brain injury, represent a major cause of disability worldwide. Despite their diverse etiologies, these conditions share common pathological mechanisms, including oxidative stress, neuroinflammation, mitochondrial dysfunction, excitotoxicity, and impaired regenerative capacity, which collectively contribute to progressive neuronal loss and limited functional recovery. Although nanotechnology has traditionally been investigated as a strategy to improve drug delivery to the CNS, growing evidence indicates that many nanomaterials possess intrinsic biological activities that extend beyond their role as passive carriers. In this review, we summarize the principal classes of neuroprotective nanomaterials and discuss the molecular and cellular mechanisms through which they modulate oxidative stress, inflammation, apoptosis, neurogenesis, synaptic plasticity, and tissue regeneration. We further examine their applications in neurodegenerative diseases, ischemic stroke, traumatic brain injury, and peripheral nerve injury, highlighting their emerging role in bridging neuroprotection and neurorepair. Importantly, we propose a conceptual shift from the conventional material-based classification of nanomaterials toward a function-based framework that categorizes nanotherapeutics according to their predominant biological activities, including antioxidant, anti-inflammatory, anti-apoptotic, neurogenic, synaptogenic/neuroplastic, regenerative, and multifunctional effects. Finally, we discuss the major challenges associated with clinical translation and future perspectives for the development of next-generation nanotherapeutics. This function-based perspective may provide a useful framework for guiding the rational design of innovative strategies aimed at promoting functional recovery following CNS injury.

## 1. Introduction

Neurological disorders and injuries affecting the CNS, including neurodegenerative diseases, ischemic stroke, and traumatic brain injury (TBI), represent a leading cause of disability and mortality worldwide [1]. Despite their diverse etiologies, these conditions share common pathological mechanisms, including oxidative stress, mitochondrial dysfunction, neuroinflammation, excitotoxicity, and progressive neuronal loss, which ultimately lead to irreversible functional impairment [2]. The limited intrinsic regenerative capacity of the adult CNS further exacerbates these outcomes, making functional recovery particularly challenging. Consequently, the development of effective neuroprotective strategies remains one of the major unmet needs in modern neuroscience.

**Figure 1:**
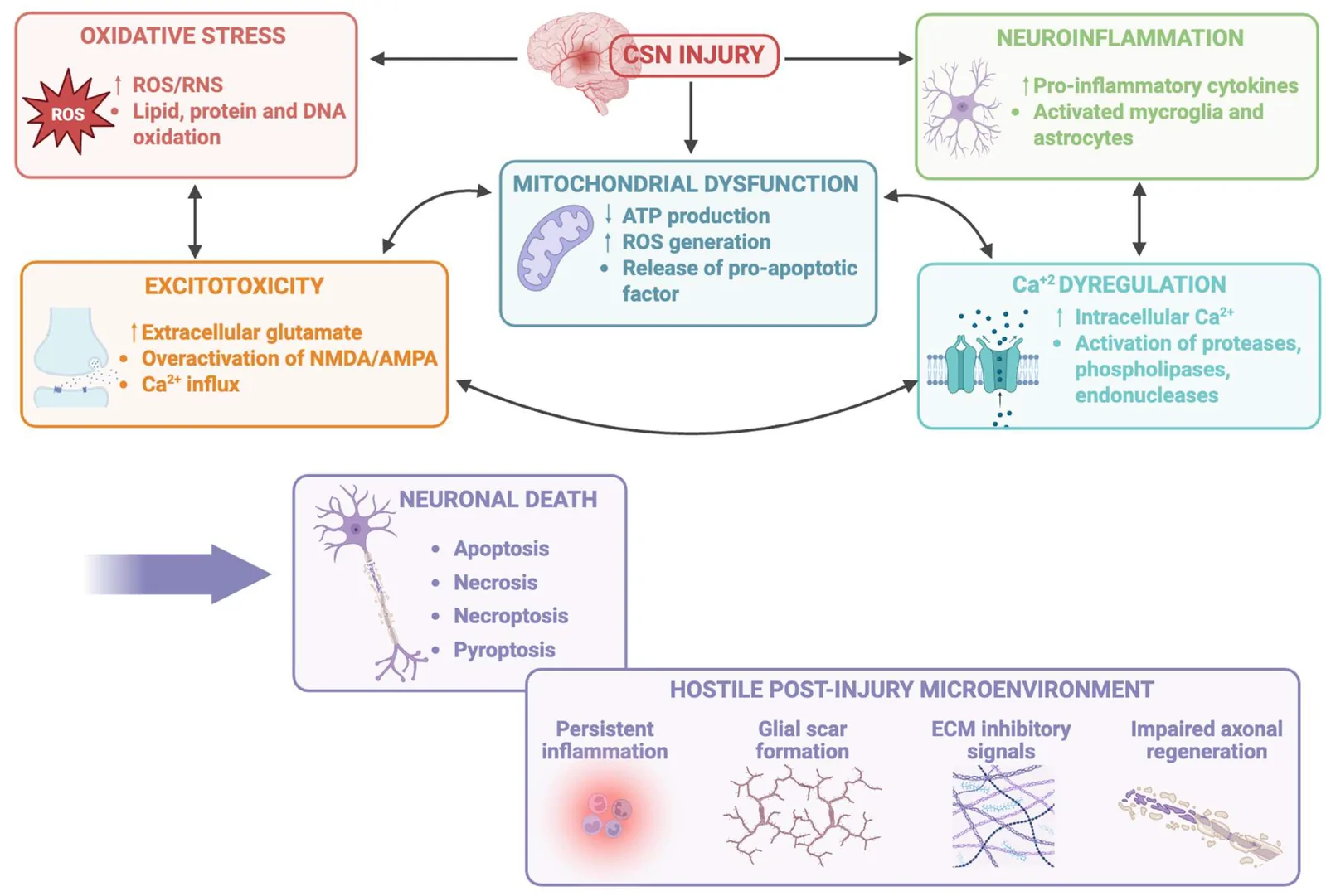
Interconnected pathological mechanisms underlying CNS injury and impaired functional recovery. CNS injury triggers a complex and self-amplifying pathological network involving oxidative stress, mitochondrial dysfunction, neuroinflammation, excitotoxicity, and calcium dysregulation. These interconnected processes promote neuronal death and contribute to the establishment of a hostile post-injury microenvironment characterized by persistent inflammation, glial scar formation, and inhibitory extracellular matrix remodeling, ultimately limiting endogenous repair and functional recovery (Created in BioRender. Colasante, M. (2026) https://BioRender.com/fgvf56p, accessed on 12 October 2026).

Over the past decades, numerous neuroprotective agents have demonstrated promising efficacy in experimental models; however, their clinical translation has been largely disappointing. Several factors contribute to this failure, including poor pharmacokinetic profiles, inadequate biodistribution, insufficient penetration across the blood–brain barrier (BBB), and the intrinsic complexity of CNS pathology [3]. Importantly, neuronal injury is not driven by a single molecular event but rather by a highly interconnected network of pathological processes that evolve dynamically over time. As a result, therapeutic approaches targeting a single pathway often fail to provide meaningful clinical benefit. The complex interplay among these mechanisms and their contribution to neuronal death and impaired tissue repair are summarized in Figure 1.

In response to these limitations, nanotechnology has emerged as a powerful platform for the treatment of neurological disorders. Nanomaterials have been extensively investigated as carriers for the delivery of drugs, proteins, nucleic acids, and other therapeutic agents to the CNS [4]. Their tunable physicochemical properties, including size, surface charge, morphology, and functionalization potential, enable improved biodistribution, enhanced BBB penetration, controlled drug release, and targeted delivery to specific cellular populations. Consequently, nanomedicine is increasingly recognized as a promising strategy to overcome many of the barriers that have traditionally limited CNS therapeutics.

However, the conventional view of nanomaterials as passive delivery vehicles is rapidly evolving. Increasing evidence suggests that many nanomaterials possess intrinsic biological activities capable of directly modulating cellular and molecular processes involved in neuronal survival and tissue repair. Depending on their composition and structural characteristics, nanomaterials can actively regulate oxidative stress [5], attenuate neuroinflammation [6], preserve mitochondrial function [7], modulate apoptosis [8], and influence neural stem cell behavior [9]. Furthermore, some nanostructured systems can support axonal growth, enhance synaptic plasticity, and promote structural features associated with neural network reconstruction. These observations indicate that nanomaterials may function not only as carriers of therapeutic agents but also as active therapeutic entities. Importantly, the traditional distinction between neuroprotection and neuroregeneration is becoming increasingly blurred. While neuroprotection aims to prevent or limit neuronal damage, successful functional recovery also requires the restoration of neural circuitry and the promotion of endogenous repair mechanisms. Therefore, contemporary therapeutic strategies are progressively shifting toward integrated approaches that simultaneously preserve neuronal viability and promote functional recovery. In this context, multifunctional nanomaterials are uniquely positioned to address both objectives, acting at multiple stages of the pathological cascade.

For clarity, several key concepts used throughout this review should be distinguished. In the present article, neuroprotection refers to interventions aimed at preventing or limiting neuronal damage and preserving cellular viability following injury or disease. Neurorepair encompasses the broader set of endogenous and therapeutic processes that contribute to the restoration of tissue structure and function after damage. Neuroregeneration is considered a component of neurorepair and primarily refers to the generation of new neural elements and the regrowth of damaged axons. Functional neurorepair is used to describe repair processes that ultimately contribute to the restoration of neural circuit function and behavioral recovery. Although neurodegenerative diseases and acute CNS injuries differ in etiology, they are considered together in this review because many neuroprotective nanomaterials target pathological mechanisms shared across these conditions. In addition, peripheral nerve injury (PNI) is included as a complementary translational model because axonal regeneration and functional recovery can often be assessed more directly than in CNS disorders, thereby providing valuable insights into the neurorepair potential of nanomaterials.

### 1.1. Novelty and Scope

Despite the growing number of reviews on neuroprotective nanomaterials, most available studies classify nanoplatforms according to their physicochemical composition or delivery characteristics. While informative, these approaches often provide limited insight into the biological functions through which nanomaterials influence neuronal survival and repair. To the best of our knowledge, no previous review has systematically organized neuroprotective nanomaterials according to their predominant biological functions. Therefore, this review proposes a function-based framework that classifies nanomaterials according to their primary biological activities, including antioxidant, anti-inflammatory, anti-apoptotic, neurogenic, synaptogenic/neuroplastic, regenerative, and multifunctional effects. Furthermore, this review integrates mechanistic insights into neuronal injury and recovery, disease-specific applications, safety considerations, translational challenges, and emerging clinical perspectives. By connecting nanomaterial function with neurobiological mechanisms and translational relevance, the proposed framework aims to provide a complementary perspective to conventional material-based classifications and to facilitate the identification of promising strategies for next-generation neuroprotective and neurorepair therapies.

### 1.2. Literature Search Strategy

Although this article was conceived as a narrative review, a structured literature search was performed to identify the most relevant studies on neuroprotective nanomaterials and neurorepair. The literature search was conducted using PubMed, Scopus, and Web of Science databases between January 2025 and July 2026. Combinations of the following keywords were used: “neuroprotective nanomaterials”, “nanomedicine”, “central nervous system injury”, “neurorepair”, “neuroregeneration”, “oxidative stress”, “neuroinflammation”, “stroke”, “traumatic brain injury”, and “neurodegenerative diseases”. Priority was given to recent original studies, high-impact reviews, translational investigations, and studies providing mechanistic insights into the biological activities of nanomaterials. Additional seminal publications were included when considered essential to contextualize the evolution of the field. The final selection was based on relevance to the objectives of this review, with particular emphasis on studies describing intrinsic neuroprotective and neuroregenerative activities of nanomaterials beyond their role as drug-delivery systems.

## 2. Classes of Neuroprotective Nanomaterials

Nanomaterials employed in CNS applications can be broadly classified into distinct categories based on their composition and physicochemical properties. These characteristics, including size, surface charge, and structural organization, strongly influence their interaction with biological systems, particularly within the complex neural microenvironment. Importantly, beyond their use as delivery platforms, many nanomaterials exhibit intrinsic biological activities that contribute directly to neuroprotection [10]. Therefore, understanding their classification is essential not only from a materials perspective but also in terms of their functional relevance in modulating key pathological processes associated with CNS injury.

### 2.1. Carbon-Based Nanomaterials

Carbon-based nanomaterials [11], including graphene, graphene oxide (GO), and carbon nanotubes (CNTs), have attracted significant attention for their potential in neurobiological applications due to their unique physicochemical and electrical properties; these materials exhibit high surface area, mechanical strength, and electrical conductivity, which enable effective interactions with neural cells [12]. In the context of neuroprotection, carbon-based nanomaterials have been shown to promote neuronal differentiation, enhance synaptic activity, and support the growth of neural stem cells [9], in particular, graphene-based materials can influence cell adhesion and cytoskeletal organization, thereby contributing to neurogenesis and network formation [13]. Additionally, their ability to interface with electrically active tissues makes them particularly suitable for modulating neuronal signaling and restoring functional connectivity. However, concerns remain regarding their potential toxicity, which is highly dependent on factors such as size, functionalization, and concentration. Careful design and surface modification are therefore crucial to maximize their biocompatibility and therapeutic potential.

### 2.2. Polymeric Nanoparticles

Polymeric nanoparticles, such as those based on poly-lactic-co-glycolic acid (PLGA), chitosan, and other biodegradable polymers, represent one of the most extensively studied classes of nanomaterials for CNS applications [14]. Their major advantages include biocompatibility, biodegradability, and the ability to encapsulate a wide range of therapeutic agents [15]. From a neuroprotective perspective, polymeric nanoparticles enable controlled and sustained release of bioactive molecules, improving their stability and bioavailability; furthermore, certain polymers such as chitosan possess intrinsic biological properties, including mucoadhesion and mild anti-inflammatory effects, which may further contribute to neuroprotection. Importantly, polymeric systems can be engineered to enhance BBB permeability through surface functionalization with targeting ligands [16]. This feature allows for improved delivery to the CNS while minimizing systemic side effects. Although primarily considered delivery systems, their design flexibility makes them valuable components in multifunctional therapeutic strategies targeting multiple pathways involved in neural damage.

### 2.3. Lipid-Based Nanocarriers

Lipid-based nanocarriers, including liposomes, solid lipid nanoparticles (SLNs), and nanostructured lipid carriers (NLCs), are widely used in CNS drug delivery due to their structural similarity to biological membranes [4]. This property facilitates their interaction with cellular membranes and enhances their ability to cross the BBB. These systems can encapsulate both hydrophilic and hydrophobic compounds, providing protection from degradation and improving pharmacokinetic profiles. In addition to their carrier function, lipid-based nanomaterials may also influence membrane dynamics and cellular uptake processes, potentially affecting neuronal survival and signaling. Moreover, their generally favorable safety profile and biocompatibility make them attractive candidates for clinical applications. However, challenges related to stability, scalability, and drug loading efficiency need to be addressed to fully exploit their therapeutic potential.

### 2.4. Inorganic Nanomaterials

Inorganic nanomaterials, such as gold nanoparticles, silica nanoparticles, and cerium oxide nanoparticles (nanoceria), offer unique physicochemical properties that can be harnessed for neuroprotective applications [17]. These materials are characterized by high stability, tunable size, and the ability to be functionalized with various ligands. Among these, cerium oxide nanoparticles are of particular interest due to their intrinsic antioxidant activity [18]. Nanoceria can cycle between different oxidation states (Ce^3+^/Ce^4+^), allowing them to scavenge reactive oxygen species and reduce oxidative stress, a key driver of neuronal damage [19]. Gold nanoparticles, on the other hand, have been explored for their anti-inflammatory properties and their ability to modulate cellular signaling pathways [20]. Despite their promising properties, concerns regarding long-term accumulation, biodegradability, and potential toxicity remain critical considerations for their clinical translation. Therefore, optimizing their design and understanding their interactions with biological systems are essential steps toward their safe application.

### 2.5. Hybrid and Multifunctional Nanomaterials

Hybrid nanomaterials combine components from different classes, such as polymeric, lipid, and inorganic systems, to create multifunctional platforms with enhanced therapeutic capabilities [21]. These systems are designed to integrate multiple properties, including targeted delivery, controlled release, and intrinsic biological activity. In the context of CNS injury, hybrid nanomaterials offer the possibility to simultaneously address multiple pathological mechanisms. As demonstrated by Chen et al., a single nanoplatform may incorporate antioxidant components to reduce oxidative stress, targeting ligands to facilitate BBB crossing, and structural elements to support neuronal growth and tissue regeneration [22]. This multifunctionality represents a significant advancement over traditional single-component systems and aligns with the need for multitarget therapeutic strategies in complex neurological disorders. Furthermore, the development of stimuli-responsive (smart) nanomaterials capable of reacting to changes in the local microenvironment, such as pH or oxidative stress levels, provides additional opportunities to enhance therapeutic efficacy and specificity.

### 2.6. Stimuli-Responsive Nanomaterials

Stimuli-responsive nanomaterials represent an advanced class of neuroprotective platforms designed to release therapeutic agents or activate biological functions in response to specific endogenous or exogenous triggers. Endogenous stimuli include variations in pH, oxidative stress levels, inflammatory mediators, and enzymatic activity commonly associated with CNS injury and neurodegenerative disorders. Exogenous stimuli, such as light, magnetic fields, ultrasound, or temperature changes, can further enable spatiotemporal control of therapeutic delivery. In the context of neuroprotection, these smart nanomaterials offer the potential to enhance treatment specificity while minimizing off-target effects. By responding to pathological microenvironments characterized by elevated reactive oxygen species, neuroinflammation, or blood–brain barrier disruption, stimuli-responsive systems may improve local drug accumulation and therapeutic efficacy. Furthermore, their ability to provide controlled and on-demand release makes them promising candidates for precision nanomedicine approaches targeting complex CNS disorders. Although most applications remain at the preclinical stage, stimuli-responsive nanoplatforms represent an emerging strategy that may facilitate the translation of neuroprotective interventions through improved targeting, safety, and therapeutic control [23].

### 2.7. Nanozymes

Nanozymes are nanomaterials with enzyme-like catalytic activities that can mimic the functions of natural antioxidant enzymes, including superoxide dismutase, catalase, and peroxidase [24]. Owing to their unique physicochemical properties, nanozymes have emerged as promising neuroprotective agents capable of modulating oxidative stress, one of the central pathological mechanisms underlying neurodegenerative disorders and acute CNS injuries. In neurological applications, nanozymes can scavenge excessive reactive oxygen species, reduce lipid peroxidation, and protect neuronal cells from oxidative damage. Beyond their antioxidant activity, certain nanozyme platforms have also demonstrated anti-inflammatory properties through the regulation of microglial activation and neuroimmune responses. These multifunctional effects may contribute to the preservation of neuronal integrity and the attenuation of progressive neurodegeneration. Compared with natural enzymes, nanozymes generally exhibit greater stability, longer circulation times, and improved resistance to environmental degradation, making them attractive candidates for therapeutic development. Although most studies remain in the preclinical stage, nanozymes represent an emerging nanotechnology-based strategy with significant potential for enhancing neuroprotection and supporting the translation of nanomedicine approaches for CNS disorders.

### 2.8. Toward a Functional Classification of Neuroprotective Nanomaterials

Although nanomaterials are traditionally classified according to their chemical composition and physicochemical characteristics, including carbon-based, polymeric, lipid-based, inorganic, and hybrid systems, this approach may not fully capture their therapeutic potential in CNS disorders. As summarized in Table 1, conventional classification provides valuable information regarding material properties, manufacturing strategies, drug-loading capabilities, and safety considerations. Such a material-centered framework has historically guided the development of nanomedicine and remains essential for understanding how different nanoplatforms interact with biological systems.

Although nanozymes may originate from different material classes, they are included as a distinct category because their enzyme-mimicking catalytic behavior represents a functionally relevant and increasingly recognized group within nanomedicine.

However, from a neurobiological perspective, nanomaterials with completely different compositions often produce similar therapeutic effects, whereas materials belonging to the same class may exert distinct biological activities depending on their size, surface chemistry, structural organization, and functionalization strategies. Therefore, classification based exclusively on composition may overlook the biological mechanisms through which nanomaterials influence neuronal survival and tissue repair. This limitation becomes particularly evident in CNS injury, where disease progression is driven by multiple interconnected pathological processes, including oxidative stress, neuroinflammation, excitotoxicity, mitochondrial dysfunction, neuronal loss, and impaired regeneration. Because several nanomaterials can simultaneously modulate these pathways, their therapeutic relevance may be more appropriately evaluated according to function rather than composition. Consequently, we propose a complementary function-based classification framework that categorizes neuroprotective nanomaterials according to their predominant biological activity within the injured nervous system. The principal functional categories, representative nanomaterials, biological targets, and potential contributions to neurorepair are summarized in Table 2.

Classification within this framework is based on the predominant biological activity consistently reported across experimental studies. The resulting framework comprises seven operational functional categories: antioxidant, anti-inflammatory, anti-apoptotic, neurogenic, synaptogenic/neuroplastic, regenerative, and multifunctional nanomaterials.

The assignment of individual nanomaterials to a specific functional category was not based exclusively on their chemical composition, therapeutic cargo, or surface functionalization, but rather on the biological activity most consistently reported across experimental studies. We recognize that nanomaterial behavior is context-dependent and may vary according to physicochemical characteristics, biological environment, disease model, and incorporated therapeutic agents. Therefore, the proposed categories should be regarded as operational and non-mutually exclusive descriptors intended to facilitate conceptual organization rather than rigid classifications. While individual nanomaterials may exhibit multiple biological activities, classification was based on the mechanism that appeared most consistently and prominently across experimental studies. Nanomaterials demonstrating multiple activities of comparable relevance were assigned to the multifunctional category.

We acknowledge that the boundaries between these categories are not absolute and that many nanomaterials may span multiple functional domains depending on their physicochemical properties and experimental context.

The framework in Table 2 highlights the transition from material-centered nanomedicine to function-driven neuroprotective strategies, classifying nanomaterials according to their antioxidant, anti-inflammatory, anti-apoptotic, neurogenic, synaptogenic, regenerative, and multifunctional properties, as well as their potential contribution to functional neurorepair and clinical translation.

**Figure 2:**
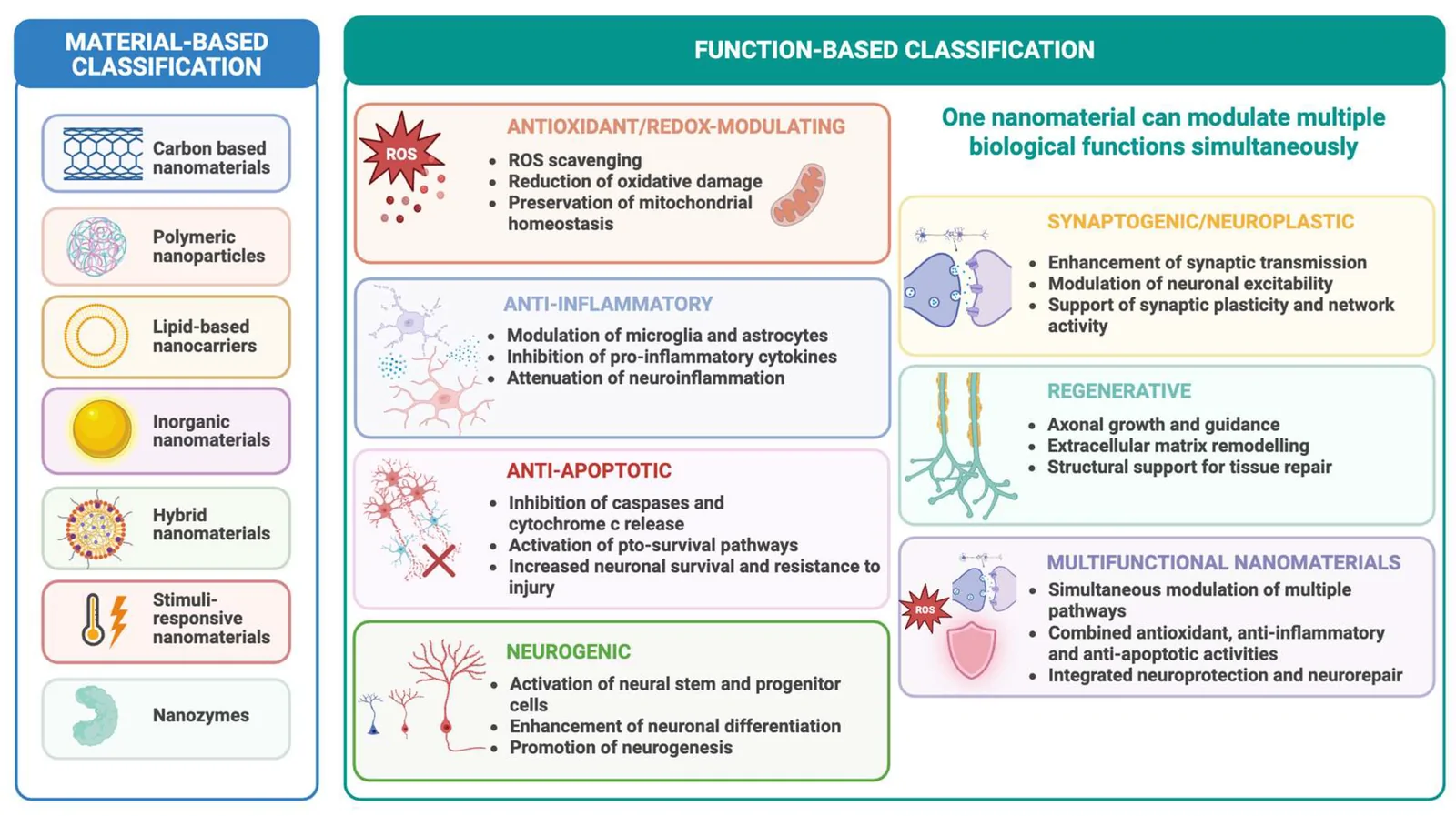
From material-based to function-based classification of neuroprotective nanomaterials. Neuroprotective nanomaterials can be classified according to their composition and physicochemical properties; however, individual nanomaterials may simultaneously modulate multiple biological processes. A function-based classification highlights their antioxidant, anti-inflammatory, anti-apoptotic, neurogenic, synaptogenic/neuroplastic, regenerative and multifunctional activities, providing a framework that better reflects their therapeutic role in CNS injury. (Created in BioRender. Colasante, M. (2026) https://BioRender.com/fgvf56p, accessed on 12 October 2026).

The conceptual relationship between the traditional material-based classification and the proposed function-based framework is schematically illustrated in Figure 2.

Importantly, this framework is not intended to replace physicochemical classification but to complement it from a therapeutic perspective. While material-based classification remains essential for understanding nanomaterial design, manufacturing, and biological interactions, functional classification provides additional insight into the mechanisms through which nanomaterials contribute to neuroprotection and neurorepair. While Table 1 answers the question “What kind of nanomaterial is being used?”, Table 2 addresses the more clinically relevant question “What biological function does the nanomaterial perform within the injured CNS?”. This perspective aligns with the growing recognition that effective treatment of CNS disorders requires multitarget strategies capable of simultaneously limiting neuronal damage and promoting tissue repair. In this context, nanomaterials should increasingly be viewed not only as delivery systems but also as active modulators of pathological and regenerative processes.

### 2.9. Antioxidant Nanomaterials

Oxidative stress represents one of the earliest and most damaging events following CNS injury [28]. Excessive production of reactive oxygen species (ROS) contributes to lipid peroxidation, protein oxidation, mitochondrial dysfunction, and activation of cell death pathways. Consequently, nanomaterials capable of directly regulating redox homeostasis constitute an important category of neuroprotective agents. Among these, cerium oxide nanoparticles (nanoceria) are particularly attractive because of their ability to cycle between Ce^3+^ and Ce^4+^ oxidation states, enabling sustained ROS scavenging. Similarly, nanozymes exhibit enzyme-mimicking activities that reproduce the functions of endogenous antioxidant enzymes, including superoxide dismutase and catalase. Graphene-derived nanomaterials have also demonstrated the ability to attenuate oxidative damage and preserve mitochondrial function. By limiting secondary injury mechanisms, antioxidant nanomaterials establish conditions that are more favorable for subsequent regenerative processes.

### 2.10. Anti-Inflammatory Nanomaterials

Persistent neuroinflammation is a major contributor to neuronal dysfunction and progressive neurodegeneration. Activated microglia and astrocytes release pro-inflammatory mediators that amplify tissue damage and inhibit endogenous repair mechanisms. Several nanomaterials possess intrinsic immunomodulatory properties. Gold nanoparticles have been shown to attenuate inflammatory signaling pathways and reduce cytokine release, while polymeric systems can influence microglial polarization and promote a more regenerative phenotype. Lipid-based nanocarriers may further contribute by facilitating localized anti-inflammatory interventions. Through the restoration of immune homeostasis, anti-inflammatory nanomaterials help to create a regenerative microenvironment that supports neuronal survival and functional recovery [6].

### 2.11. Anti-Apoptotic Nanomaterials

Apoptotic cell death is a major contributor to neuronal loss following acute and chronic neurological disorders. Several nanomaterial-based strategies have demonstrated the ability to interfere with apoptosis-related pathways by modulating mitochondrial dysfunction, reducing oxidative damage, regulating the Bcl-2/Bax ratio, and suppressing caspase activation. In experimental models, nanocarriers loaded with neurotrophic factors, peptide-based therapeutics, or anti-apoptotic agents have been shown to enhance neuronal survival and preserve tissue integrity. Within the present framework, nanomaterials were classified as anti-apoptotic when attenuation of programmed cell death represented the predominant and most consistently reported biological effect [8]. Such interventions are particularly relevant during the acute and subacute phases of CNS injury, where limiting neuronal apoptosis may contribute to improved functional outcomes.

### 2.12. Neurogenic Nanomaterials

The limited neurogenic capacity of the adult CNS remains a major challenge for functional recovery after injury. Neurogenic nanomaterials are specifically designed to enhance neural stem cell proliferation, differentiation, and maturation [9]. Graphene-based materials are among the most extensively studied examples, owing to their ability to regulate cell adhesion, cytoskeletal organization, and mechanotransduction pathways involved in neuronal differentiation. Conductive biomaterials further provide electrical and biophysical cues that mimic features of the native neural environment. By promoting endogenous neurogenesis, these nanomaterials contribute directly to the replenishment of damaged neuronal populations.

### 2.13. Synaptogenic and Neuroplastic Nanomaterials

Functional recovery after neurological injury depends not only on neuronal survival but also on the restoration of neural connectivity and network organization. A growing number of nanomaterials have been reported to promote neurite outgrowth, synapse formation, and activity-dependent plasticity through the modulation of intracellular signaling pathways and neurotrophic support. Conductive nanomaterials, graphene-based platforms, and engineered delivery systems capable of sustained neurotrophic factor release have demonstrated the ability to enhance synaptic remodeling and facilitate neural circuit reorganization. In the present classification, nanomaterials were assigned to the synaptogenic/neuroplastic category when enhancement of synaptic connectivity or neuroplastic adaptation represented the primary mechanism underlying their neuroprotective or neurorestorative effects [13].

### 2.14. Regenerative Nanomaterials

While neurogenesis is important, successful recovery also requires reconstruction of damaged neural networks. Neuroregenerative nanomaterials therefore focus on enhancing axonal growth, synaptic reconnection, and tissue remodeling [29]. Representative examples include electrospun nanofibers, hydrogel-based scaffolds, and nerve guidance conduits. These systems provide structural support for cell migration and axonal elongation while simultaneously delivering biochemical signals that promote regeneration. Unlike conventional neuroprotective interventions, neuroregenerative nanomaterials actively participate in tissue reconstruction and network reorganization.

### 2.15. Multifunctional and Intelligent Nanomaterials

The multifactorial nature of CNS disorders increasingly drives the development of multifunctional nanoplatforms capable of targeting several pathological mechanisms simultaneously [21]. Hybrid nanomaterials combine features derived from distinct material classes to achieve integrated antioxidant, anti-inflammatory, neurogenic, and regenerative effects. More recently, intelligent and stimuli-responsive systems have emerged as advanced therapeutic platforms capable of responding dynamically to disease-associated signals such as oxidative stress, altered pH, or inflammatory mediators. These systems may offer unprecedented opportunities for personalized and adaptive neuroprotective interventions.

### 2.16. From Material-Centered Nanomedicine to Function-Driven Neurorepair

Taken together, the frameworks presented in Table 1 and Table 2 illustrate the ongoing evolution of neuroprotective nanomedicine. Traditional classification remains essential for understanding material design and physicochemical behavior, whereas functional classification more effectively captures therapeutic relevance and biological activity. This conceptual shift from material-centered nanomedicine toward function-driven neurorepair represents the central perspective of the present review. Within this framework, nanomaterials are viewed as active modulators of pathological and regenerative processes involved in CNS injury.

To further illustrate the experimental basis underlying the proposed functional categories, representative in vitro and in vivo studies are summarized in Table 3. The following section therefore examines the principal mechanisms through which antioxidant, anti-inflammatory, anti-apoptotic, neurogenic, synaptogenic/neuroplastic, regenerative, and multifunctional nanomaterials exert their effects, providing the mechanistic foundation for the proposed function-based classification.

Table 3 summarizes the principal mechanisms of action, representative nanomaterials, experimental evidence, and translational relevance associated with each functional category. It highlights the varying levels of support currently available from in vitro studies, preclinical models, and translational investigations, providing a critical overview of the maturity of the evidence across the field.

It is important to recognize that the evidence supporting nanomaterial-mediated neurorepair spans different experimental levels and should therefore be interpreted accordingly. Most reported benefits derive from in vitro studies and small-animal models demonstrating molecular, cellular, and histological changes, including reductions in oxidative stress, modulation of inflammatory pathways, attenuation of apoptosis, and promotion of neural differentiation. Evidence supporting structural regeneration, such as axonal growth and tissue remodeling, is increasingly available in preclinical models. In contrast, demonstrations of electrophysiological recovery remain comparatively limited, while behavioral and functional improvements have been reported predominantly in animal studies. Consequently, molecular and structural indicators of neurorepair should not be interpreted as direct evidence of functional restoration unless supported by dedicated electrophysiological, behavioral, or clinical assessments. This distinction is reflected in Table 3, which summarizes the current level of supporting evidence associated with each functional category.

## 3. Mechanisms of Neuroprotection Mediated by Nanomaterials

**Figure 3:**
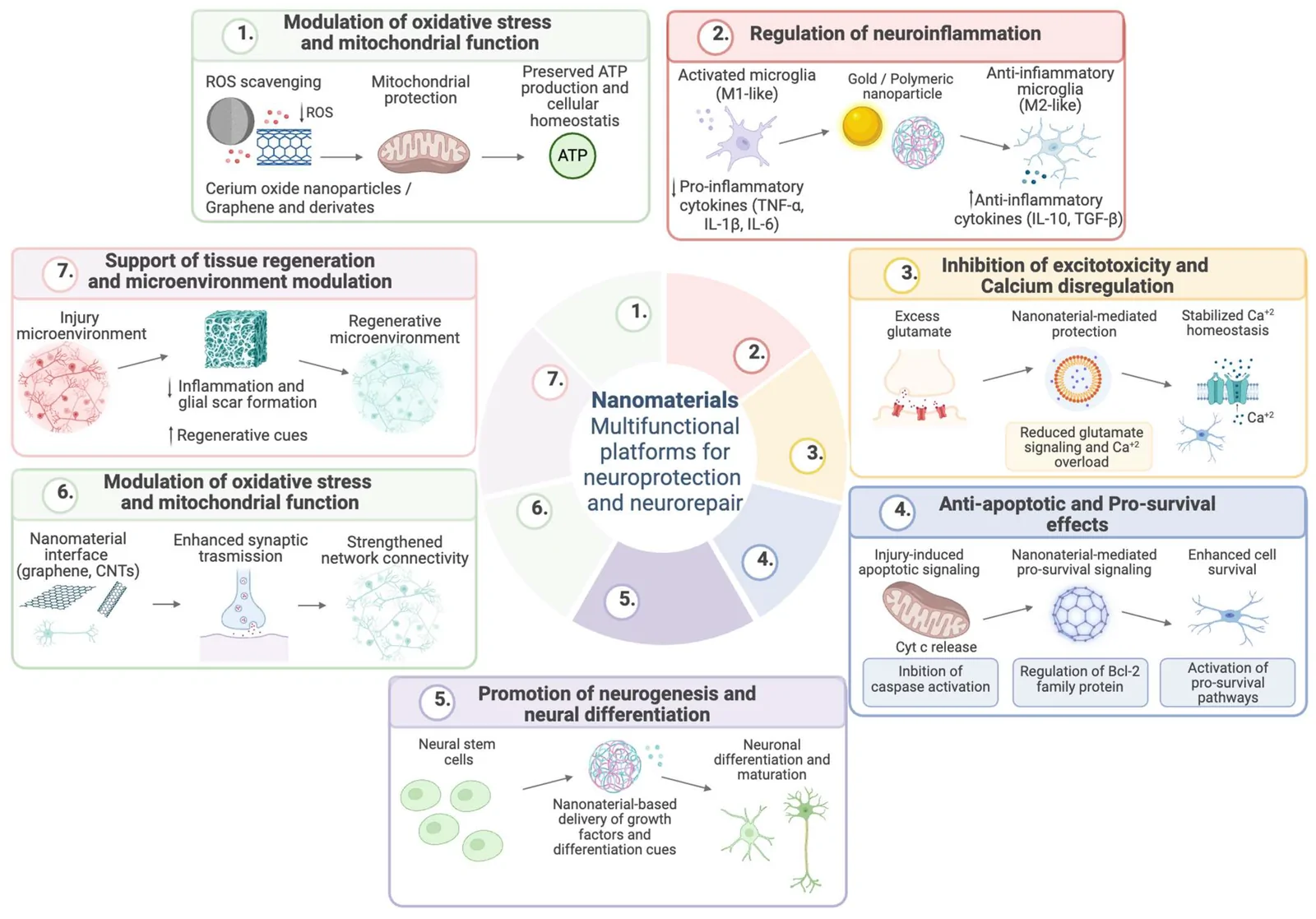
Multifunctional mechanisms underlying nanomaterial-mediated neuroprotection and neurorepair. Nanomaterials can modulate multiple interconnected pathological and regenerative processes involved in CNS injury. These include (**1**) the reduction in oxidative stress and preservation of mitochondrial function, (**2**) the regulation of neuroinflammatory responses, (**3**) the attenuation of excitotoxicity and calcium dysregulation, (**4**) the inhibition of apoptotic pathways and promotion of cell survival, (**5**) promotion of neurogenesis and neuronal differentiation, (**6**) synaptic plasticity and network reconstruction, and (**7**) tissue regeneration through modulation of the post-injury microenvironment. Collectively, these multifunctional activities highlight the potential of nanomaterials to extend beyond conventional drug delivery systems and act as active modulators of neuroprotection and neurorepair. (Created in BioRender. Colasante, M. (2026) https://BioRender.com/fgvf56p, accessed on 12 October 2026).

Given the interconnected nature of CNS injury, nanomaterials may influence multiple pathological pathways simultaneously. Traditional therapeutic approaches have largely focused on single mechanisms, often with limited clinical success. In contrast, nanomaterials, due to their unique physicochemical properties, are emerging as multifunctional platforms capable of modulating several biological processes at once. Beyond their role as drug delivery systems, nanomaterials can actively interact with neural cells and the surrounding microenvironment, influencing key mechanisms involved in neuroprotection and neurorepair [30] (Figure 3).

### 3.1. Modulation of Oxidative Stress and Mitochondrial Function

Oxidative stress and mitochondrial dysfunction represent central events in CNS injury and neurodegeneration [31]. The excessive production of ROS leads to lipid peroxidation, protein oxidation, and DNA damage, ultimately compromising neuronal survival. Mitochondria, which are both a major source and target of ROS, play a crucial role in maintaining cellular homeostasis, and their dysfunction amplifies oxidative damage. Nanomaterials can act as direct regulators of redox balance through different mechanisms. Certain inorganic nanomaterials, such as cerium oxide nanoparticles, exhibit intrinsic antioxidant properties due to their ability to switch between oxidation states, allowing continuous scavenging of ROS [32]. Similarly, carbon-based nanomaterials, including graphene and its derivatives, have been reported to reduce oxidative stress and preserve mitochondrial integrity [33]. In addition, nanocarriers can deliver mitochondria-targeted therapeutics, enhancing their effectiveness and protecting mitochondrial function [34]. By reducing ROS levels and preserving mitochondrial homeostasis, nanomaterials contribute to the stabilization of cellular metabolism, improved ATP production, and prevention of downstream cell death pathways.

Together, these findings support the classification of antioxidant nanomaterials as active regulators of cellular redox homeostasis. By directly scavenging reactive oxygen species and preserving mitochondrial function, these systems contribute to both acute neuroprotection and long-term neurorepair.

### 3.2. Regulation of Neuroinflammation

Neuroinflammation, driven by activated microglia and astrocytes, plays a critical role in the progression of CNS injury [35]. Persistent activation of glial cells leads to the release of pro-inflammatory cytokines and exacerbates neuronal damage. Importantly, neuroinflammation does not occur in isolation but is closely interconnected with other pathological processes, including oxidative stress, BBB dysfunction, and neuronal hyperexcitability. Pro-inflammatory mediators can promote reactive oxygen species production, which further damages neuronal and vascular structures, while BBB disruption facilitates peripheral immune cell infiltration, amplifying inflammatory cascades. These events collectively contribute to neuronal dysfunction and perpetuate injury progression. Nanomaterials have emerged as promising modulators of neuroinflammatory responses. Several nanoparticle systems have been shown to reduce the expression of pro-inflammatory mediators and promote a shift in microglial activation from a pro-inflammatory (M1-like) to an anti-inflammatory (M2-like) phenotype [36]. For instance, gold nanoparticles and polymer-based systems can attenuate inflammatory signaling pathways, including NF-κB, thereby decreasing cytokine release [37]. Moreover, targeted delivery of anti-inflammatory agents using nanocarriers further enhances therapeutic efficacy while minimizing systemic effects. By modulating glial activation and restoring immune balance, nanomaterials contribute to a microenvironment that is more permissive for neuronal survival and regeneration. Given the interconnected nature of CNS injury, modulation of neuroinflammation may also indirectly attenuate oxidative stress, preserve BBB integrity, and reduce secondary neuronal dysfunction.

Overall, these observations identify anti-inflammatory nanomaterials as an important functional category capable of reshaping the post-injury microenvironment and facilitating endogenous repair mechanisms.

### 3.3. Inhibition of Excitotoxicity and Calcium Dysregulation

Excitotoxicity, primarily mediated by excessive glutamate signaling and calcium influx, is a major contributor to neuronal damage in CNS injury. The overactivation of glutamate receptors leads to intracellular Ca^2+^ overload, which in turn triggers mitochondrial dysfunction, ROS production, and activation of degradative enzymes. Although less extensively explored than other mechanisms, emerging evidence suggests that nanomaterials can mitigate excitotoxic damage by modulating cellular microenvironments and stabilizing neuronal membranes. Some nanocarriers can regulate glutamate levels indirectly through improved metabolic support or by delivering neuroprotective agents that inhibit receptor overactivation [38]. Additionally, nanomaterials that preserve mitochondrial function contribute to buffering intracellular calcium levels and reducing downstream toxicity [39]. These effects highlight the potential of nanotechnology to intervene in early stages of neuronal injury and prevent the amplification of excitotoxic cascades.

Although still an emerging field, current evidence suggests that certain nanomaterials may indirectly mitigate excitotoxic damage by preserving metabolic homeostasis and limiting downstream calcium-dependent toxicity.

### 3.4. Anti-Apoptotic and Pro-Survival Effects

The activation of apoptotic pathways represents a common endpoint of multiple pathological processes in CNS injury. Mitochondrial dysfunction and oxidative stress trigger the release of cytochrome c and the activation of caspases, leading to programmed cell death. Nanomaterials can exert anti-apoptotic effects both directly and indirectly [40]. In particular, nanocarrier-based systems can deliver therapeutic agents that inhibit caspase activation or modulate the balance between pro-apoptotic and anti-apoptotic proteins, including members of the Bcl-2 family. Such regulatory effects have been extensively described in non-neural systems [41,42] and are increasingly being reported in experimental models of CNS injury. Furthermore, several nanomaterials have been shown to activate pro-survival signaling pathways, such as PI3K/Akt and MAPK, thereby enhancing neuronal resistance to injury and promoting cell survival [43]. Collectively, these actions contribute to limiting neuronal loss and preserving tissue integrity. These findings support the inclusion of anti-apoptotic nanomaterials within the proposed functional framework, as their neuroprotective effects help maintain the cellular foundation necessary for subsequent repair and functional recovery.

### 3.5. Promotion of Neurogenesis and Neural Differentiation

In addition to preventing neuronal loss, effective neuroprotective strategies should promote the generation of new neurons and the restoration of neural circuits. However, endogenous neurogenesis in the adult CNS is limited and often insufficient to compensate for injury. Nanomaterials, particularly carbon-based materials and engineered scaffolds, have demonstrated the ability to support neural stem cell proliferation and differentiation. Graphene-based substrates, for example, provide a favorable microenvironment for stem cell adhesion and neuronal lineage commitment [44]. Likewise, polymeric and hybrid nanostructures can deliver growth factors and differentiation cues, enhancing neurogenic processes [45]. By promoting neuronal differentiation and maturation, nanomaterials may support cellular replacement processes and favor network remodeling following injury. Neurogenic nanomaterials therefore represent a distinct functional class capable of actively promoting neuronal replacement and enhancing endogenous regenerative capacity.

### 3.6. Enhancement of Synaptic Plasticity and Network Reconstruction

Synaptic plasticity is essential for functional recovery following CNS injury. The formation of new synaptic connections and the strengthening of existing ones are critical processes underlying learning, memory, and neural repair. Nanomaterials can influence synaptic plasticity through both biochemical and biophysical mechanisms. Electrically conductive materials, such as graphene and carbon nanotubes, can interface with neuronal networks [46] and modulate electrical activity, potentially enhancing synaptic transmission. Additionally, nanomaterials that support neuronal growth and alignment facilitate the formation of organized neural networks [47]. These effects may facilitate connectivity restoration and network integration, moving beyond simple neuroprotection toward active neuroregeneration.

By facilitating network reorganization and synaptic remodeling, these platforms extend the concept of neuroprotection beyond cell survival toward restoration of functional connectivity.

### 3.7. Support of Tissue Regeneration and Microenvironment Modulation

The hostile microenvironment following CNS injury, characterized by inflammation, oxidative stress, and glial scar formation, represents a major barrier to regeneration. Effective therapeutic approaches must therefore not only target individual cellular processes but also modify the overall tissue environment. Nanomaterials, particularly in the form of three-dimensional scaffolds, provide structural support for tissue regeneration and can mimic the extracellular matrix. These scaffolds promote cell adhesion, migration, and axonal growth, facilitating network reconstruction [48]. In addition, they can be functionalized with bioactive molecules to modulate the local microenvironment, reducing inhibitory signals and enhancing regenerative cues [49]. By reshaping the post-injury environment, nanomaterials create conditions that are more conducive to repair and functional recovery.

Collectively, regenerative nanomaterials contribute to tissue reconstruction and create conditions that may support functional recovery, making them central components of next-generation neurorepair strategies.

## 4. Applications of Neuroprotective Nanomaterials in CNS Injury

The multifunctional nature of neuroprotective nanomaterials has enabled their application across a broad spectrum of neurological disorders. Unlike conventional therapeutic approaches, which generally target individual pathological mechanisms, nanomaterials can simultaneously modulate oxidative stress, inflammation, mitochondrial dysfunction, neuronal survival, and tissue regeneration. However, the strength of evidence supporting these applications varies considerably among disease settings. While preclinical studies have generated promising results, significant challenges remain before these technologies can be translated into clinically effective therapies.

### 4.1. Neurodegenerative Diseases

Neurodegenerative disorders such as Alzheimer’s disease (AD) and Parkinson’s disease (PD) represent attractive targets for nanomedicine due to their multifactorial pathogenesis and the persistent challenge of achieving effective CNS drug delivery. Current evidence indicates that neuroprotective nanomaterials may influence several disease-driving mechanisms simultaneously, including oxidative stress, mitochondrial dysfunction, protein aggregation, and chronic neuroinflammation [50].

In AD models, nanoparticle-based systems have been reported to reduce amyloid-β aggregation, attenuate oxidative damage, and modulate neuroinflammatory responses. Similarly, in PD, several nanomaterial formulations have demonstrated the ability to protect dopaminergic neurons, preserve mitochondrial function, and improve the delivery of neuroprotective compounds. Graphene-based materials and engineered polymeric systems have additionally shown potential to support neuronal survival and neurogenic responses [51,52,53].

Despite these encouraging findings, most available evidence remains limited to in vitro studies and small-animal models. The biological complexity of neurodegenerative diseases, which often evolve over decades, cannot be fully recapitulated in experimental systems. Furthermore, many studies primarily evaluate surrogate endpoints, such as biomarker reduction or histological improvement, without demonstrating meaningful long-term functional recovery. Consequently, although nanomaterials show considerable promise in modifying disease-associated pathways, their capacity to alter disease progression in human patients remains uncertain.

Collectively, current evidence suggests that the greatest advantage of neuroprotective nanomaterials in neurodegenerative disorders may reside in their multitarget activity rather than in improved drug delivery alone. Nevertheless, robust translational studies capable of linking mechanistic effects to clinically relevant outcomes are still needed.

### 4.2. Ischemic Stroke

Among CNS disorders, ischemic stroke represents one of the most compelling applications for neuroprotective nanomedicine. The rapid onset of oxidative stress, neuroinflammation, excitotoxicity, and neuronal death generates multiple therapeutic targets that can potentially be addressed by multifunctional nanomaterials [52].

Several nanoparticle systems have demonstrated efficacy in reducing acute ischemic injury through antioxidant and anti-inflammatory mechanisms. In particular, nanoceria and nanozyme-based systems have shown remarkable ROS-scavenging activity, mitigating secondary injury cascades that contribute substantially to infarct expansion. Polymeric and lipid-based nanocarriers have additionally improved the delivery of neuroprotective agents to ischemic tissue. Moreover, increasing attention has been directed toward nanomaterials capable of promoting angiogenesis, neurogenesis, and tissue remodeling during the recovery phase [54,55,56]. However, the apparent success observed in experimental stroke models should be interpreted cautiously. Stroke research has historically been characterized by a large number of neuroprotective interventions that performed well in preclinical models but failed during clinical translation. Many experimental studies are conducted under highly controlled conditions that do not adequately reflect patient heterogeneity, comorbidities, age-related changes, or treatment delays commonly encountered in clinical practice. An important emerging concept is that nanomaterials may contribute not only to acute neuroprotection but also to post-stroke recovery by promoting angiogenesis, neurogenesis, and tissue remodeling. If confirmed in translational studies, this dual neuroprotective and regenerative activity could represent a major advantage over traditional therapies focused exclusively on acute infarct reduction.

Collectively, current evidence suggests that the major therapeutic advantage of nanomaterials in ischemic stroke is not limited to improved drug delivery but rather resides in their ability to simultaneously modulate oxidative stress, inflammation, angiogenesis, and tissue remodeling.

### 4.3. Traumatic Brain Injury

TBI is characterized by a primary mechanical insult followed by a prolonged secondary injury phase involving oxidative stress, neuroinflammation, mitochondrial dysfunction, BBB disruption, and progressive neurodegeneration. This extended pathological window makes TBI particularly attractive for intervention with multifunctional nanotherapeutic platforms.

A growing body of evidence indicates that antioxidant nanoparticles, nanozymes, and hybrid nanomaterials can attenuate secondary injury mechanisms following TBI. Experimental studies have demonstrated reductions in oxidative damage, modulation of inflammatory responses, preservation of mitochondrial function, and support of regenerative pathways. In parallel, scaffold-based nanomaterials have shown potential for promoting axonal regeneration and neural tissue reconstruction [57,58]. Nevertheless, the translational landscape remains challenging. Current evidence is almost entirely preclinical, and substantial variability exists among experimental models, injury severities, and therapeutic protocols. Furthermore, the long-term effects of nanomaterial administration following TBI remain poorly understood, particularly with regard to biodistribution, persistence within neural tissues, and safety.

Overall, current evidence indicates that the value of nanotechnology in TBI extends beyond mitigation of acute damage. By simultaneously targeting oxidative stress, inflammation, mitochondrial dysfunction, and regenerative pathways, nanomaterials may provide a comprehensive therapeutic strategy aimed at both limiting secondary injury and promoting long-term neurorepair.

### 4.4. Peripheral Nerve Injury as a Translational Model for Neurorepair

Although peripheral nerve injury (PNI) falls outside the CNS, it provides an important translational model for evaluating the regenerative potential of neuroprotective nanomaterials. Compared with the CNS, the peripheral nervous system exhibits a greater intrinsic regenerative capacity, allowing the biological effects of nanomaterials on tissue repair and functional recovery to be more readily assessed.

A key advantage of PNI models is the transient disruption of the blood-nerve barrier, which facilitates the accumulation of nanomaterials at sites of injury and allows more direct investigation of their biological activities. Nanostructured scaffolds, conductive biomaterials, and hybrid nanoplatforms have been explored extensively for their ability to guide axonal growth, promote Schwann cell activity, and enhance neural regeneration. Graphene-based materials have additionally demonstrated promising effects on stem cell differentiation and neural network formation [9,59].

Importantly, PNI research has highlighted one of the most significant emerging concepts in regenerative nanomedicine: nanomaterials can contribute directly to regenerative processes and functional recovery. This distinction is particularly relevant to the central hypothesis of the present review, namely that neuroprotective nanomaterials should increasingly be viewed as therapeutic entities with intrinsic biological activity.

However, caution is warranted when extrapolating findings from peripheral nerve regeneration to CNS repair. The regenerative environments of the PNS and CNS differ substantially, particularly with regard to glial responses, inhibitory signaling, and intrinsic regenerative capacity. Therefore, although PNI provides valuable proof-of-concept evidence for nanomaterial-mediated neurorepair, further studies are required to determine the extent to which these findings can be translated to CNS disorders.

Collectively, PNI models provide a useful experimental platform for investigating the regenerative potential of neuroprotective nanomaterials and may serve as an important bridge between mechanistic studies and future clinical translation.

**Figure 4:**
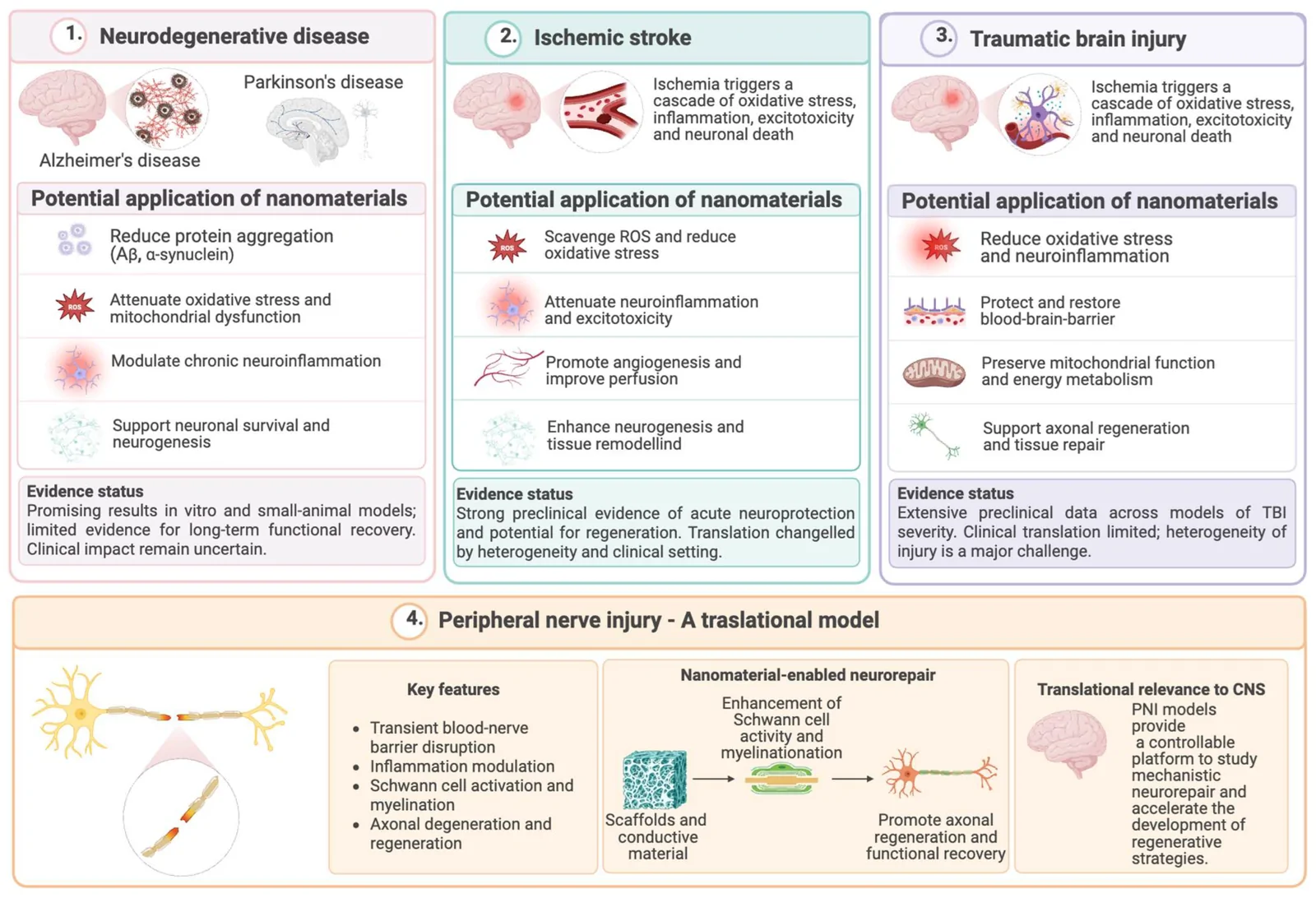
Applications of neuroprotective nanomaterials across neurological disorders. Nanomaterials may support neuroprotection and tissue repair in neurodegenerative disease, ischemic stroke, and traumatic brain injury by targeting multiple pathological processes. Peripheral nerve injury provides a complementary translational model for investigating nanomaterial-enabled neurorepair, including Schwann cell activation, myelination, axonal regeneration, and functional recovery. (Created in BioRender. Colasante, M. (2026) https://BioRender.com/fgvf56p, accessed on 12 October 2026).

Overall, the available evidence highlights the broad therapeutic potential of neuroprotective nanomaterials across multiple neurological conditions. However, important differences remain in the maturity of the available evidence, translational readiness, and clinical applicability of the various nanomaterial platforms. A critical overview of the current state of the field is summarized in Table 4 and Figure 4.

Table 4 emphasizes the existing gap between promising preclinical findings and clinical implementation, while identifying areas in which multifunctional and function-driven nanotherapeutic strategies may provide the greatest potential for future neurorepair interventions.

## 5. Challenges in Neuroprotective Nanomedicine

Despite the remarkable progress achieved in the development of nanomaterials for neuroprotection, several critical challenges still limit their clinical translation. While preclinical studies have demonstrated promising results across multiple models of CNS injury, the successful implementation of nanomaterial-based therapies in clinical settings remains limited. Addressing these challenges is essential to fully exploit the potential of nanotechnology in neuroprotection and neurorepair.

### 5.1. Biological Barriers and Targeting Limitations

One of the main challenges in CNS drug delivery is the presence of the BBB, which strictly regulates the passage of molecules into the brain. Although nanomaterials have shown improved ability to cross the BBB compared to conventional therapeutics, their transport remains highly variable and dependent on physicochemical properties such as size, charge, and surface functionalization [61]. Moreover, achieving precise targeting within specific brain regions or cell populations remains a significant challenge. Off-target distribution may result in reduced therapeutic efficacy and potential side effects. The development of advanced targeting strategies, including ligand-functionalized nanoparticles [62] and receptor-mediated transport systems [63], represents a promising approach to enhance selectivity and improve therapeutic outcomes.

It should also be recognized that BBB penetration is not a universal requirement across all neuroprotective nanomedicine applications. Depending on the anatomical location and pathological context, nanomaterials may encounter distinct barrier systems, including the BBB, the blood-spinal cord barrier (BSCB), or injury-altered barriers characterized by increased permeability and disrupted tissue architecture. Moreover, tissue accumulation, cellular uptake, barrier penetration, and therapeutic payload delivery represent distinct biological and therapeutic endpoints and should not be considered interchangeable. Increased accumulation within neural tissue does not necessarily indicate efficient intracellular delivery or therapeutic efficacy. Following CNS injury, transient alterations in barrier integrity may facilitate nanomaterial access to target tissues; however, enhanced distribution alone does not guarantee effective target engagement or therapeutic benefit. These considerations highlight the importance of distinguishing biodistribution, cellular targeting, and biological efficacy when evaluating neuroprotective nanomaterials.

### 5.2. Toxicity, Biocompatibility, and Long-Term Safety

The safety profile of nanomaterials remains a major concern for their clinical application; while many nanomaterials are designed to be biocompatible, their interactions with biological systems can lead to unexpected effects, including oxidative stress, inflammation, and immune activation. In particular, inorganic nanomaterials may accumulate in tissues due to limited biodegradability, raising concerns about long-term toxicity. Even materials generally considered safe, such as polymeric or lipid-based systems, can exhibit dose-dependent adverse effects. A comprehensive evaluation of nanomaterial toxicity, including long-term studies and standardized assessment protocols, is therefore essential.

Future research should focus on the design of biodegradable and self-eliminating nanomaterials to reduce long-term accumulation and facilitate clearance. Nevertheless, biodegradability should be considered only one component of nanomaterial safety, which is also influenced by multiple physicochemical and biological factors [64].

Importantly, the safety profile of nanomaterials cannot be uniformly generalized across different nanoplatforms, as their biological behavior is highly dependent on physicochemical characteristics, including particle size, morphology, surface charge, surface functionalization, aggregation state, dose, and route of administration. These parameters may substantially influence biodistribution, cellular uptake, degradation kinetics, tissue accumulation, and clearance, thereby affecting both therapeutic performance and safety profiles. Moreover, following exposure to biological fluids, the formation of a protein corona may alter nanoparticle physicochemical properties and modify their biological interactions, potentially affecting their interactions with immune cells, neural cells, and biological barriers. Consequently, safety assessments should be conducted on a case-by-case basis, as toxicity and biocompatibility are determined by the complex interplay of physicochemical properties, biological interactions, and exposure conditions rather than by nanomaterial class alone.

Particular attention should be devoted to the long-term biological consequences of nanoparticle exposure within the nervous system. Depending on their composition, biodistribution, and biodegradability, certain nanomaterials may be associated with neurotoxicity, immunogenic responses, prolonged microglial activation, or off-target accumulation in neural and peripheral tissues. Furthermore, incomplete degradation and prolonged retention within the brain may raise concerns regarding chronic exposure, particularly for inorganic or poorly biodegradable nanomaterials. It is also important to recognize that the biological effects of nanomaterials are often context-dependent. Although many nanomaterials have demonstrated antioxidant and neuroprotective properties, experimental evidence suggests that, under specific conditions, including inappropriate physicochemical characteristics, elevated concentrations, or prolonged exposure, some nanomaterials may contribute to reactive oxygen species generation, inflammatory responses, or cellular stress. Consequently, comprehensive safety assessments encompassing biodistribution, biodegradation, clearance, immunological responses, neuroinflammatory effects, and long-term toxicity remain essential for the successful clinical translation of neuroprotective nanomedicine.

Importantly, the acceptable safety profile may vary according to the intended neurological application. Acute neuroprotective interventions may tolerate different risk-benefit considerations than chronic therapies designed for long-term administration in neurodegenerative disorders, where prolonged exposure and cumulative effects become particularly relevant.

### 5.3. Complexity of CNS Pathology and Translational Gaps

The multifactorial nature of CNS disorders represents a fundamental obstacle to therapeutic success. While nanomaterials are uniquely suited to modulate multiple pathways simultaneously, translating these complex interactions from preclinical models to humans remains challenging. Animal models often fail to fully recapitulate the heterogeneity and progression of human CNS diseases. As a result, many promising neuroprotective strategies show limited efficacy in clinical trials. This translational gap highlights the need for more predictive experimental models, including human-derived organoids and advanced in vitro systems [65]. In addition, treatment timing is a critical factor, particularly in acute conditions such as stroke and traumatic brain injury, where therapeutic windows are narrow. The optimization of administration routes, dosing regimens, and treatment timing will be crucial for clinical success.

### 5.4. Standardization, Scalability, and Regulatory Challenges

Another major limitation in the field of nanomedicine is the lack of standardization in the design, characterization, and evaluation of nanomaterials. Variability in synthesis methods and physicochemical properties makes it difficult to compare results across studies and compromises reproducibility. Furthermore, the large-scale production of nanomaterials with consistent quality remains a significant challenge. Issues related to batch-to-batch variability, stability, and cost-effectiveness must be addressed before clinical translation can be achieved. Regulatory frameworks for nanomedicine are still evolving, and the complex nature of nanomaterials poses challenges for safety assessment and approval. Clear guidelines and standardized protocols are needed to facilitate the translation of nanotechnology-based therapies from bench to bedside.

### 5.5. Toward Intelligent and Multifunctional Nanotherapeutics

Future developments in neuroprotective nanomedicine are likely to focus on the design of intelligent and multifunctional nanomaterials capable of responding dynamically to the pathological environment [66]. Stimuli-responsive systems, which can release therapeutic agents in response to specific triggers such as oxidative stress, pH changes, or enzymatic activity, represent a promising strategy to increase precision and efficacy. In addition, the integration of multiple functionalities within a single platform, such as antioxidant activity, anti-inflammatory effects, and regenerative support, aligns with the need for multitarget therapeutic approaches in CNS disorders. Hybrid nanomaterials and modular systems offer the possibility to tailor treatments according to specific pathological conditions [67]. These advances may significantly enhance the ability of nanomaterials to act as active neuroprotective agents, rather than passive delivery systems.

### 5.6. Integration with Advanced Therapeutic Strategies

Another promising direction is the combination of nanomaterials with emerging therapeutic approaches, including stem cell therapy, gene therapy, and RNA-based technologies [68]. Nanomaterials can improve the delivery, stability, and targeting of these therapies, thereby enhancing their efficacy. For example, nanostructured scaffolds can support stem cell survival and differentiation [69], while nanoparticle-based systems can deliver genetic material to modulate gene expression in specific cell populations [70]. These integrated approaches have the potential to promote both neuroprotection and functional neurorepair. Furthermore, the implementation of personalized nanomedicine, based on patient-specific characteristics and disease profiles, may represent a new frontier in CNS therapy [71].

### 5.7. Clinical Translation and Human Evidence

Despite the remarkable advances achieved in preclinical models, the clinical translation of neuroprotective nanomaterials remains limited. While nanomedicine has successfully entered clinical practice in several therapeutic areas, including oncology and vaccine delivery, its application in the treatment of central nervous system disorders is still at an early stage [72]. One of the principal challenges is the blood–brain barrier, which continues to restrict the efficient accumulation of therapeutic nanomaterials within the brain parenchyma despite substantial progress in nanoparticle engineering.

To date, several nanomedicine platforms have received regulatory approval for non-neurological indications, demonstrating the clinical feasibility of nanoparticle-based therapeutics and providing an important foundation for future CNS applications [73,74]. However, only a limited number of neuro-targeted nanotherapeutic systems have progressed to advanced clinical evaluation. This discrepancy highlights the unique complexity of CNS disorders, which require not only effective delivery across biological barriers but also precise modulation of highly interconnected pathological pathways.

Another important consideration is the gap between preclinical efficacy and clinical performance. Many nanomaterials have demonstrated promising neuroprotective effects in rodent models of neurodegenerative diseases, stroke, and traumatic brain injury, but these benefits often fail to translate into human studies due to interspecies differences, disease heterogeneity, variability in treatment timing, and challenges associated with large-scale manufacturing and reproducibility [75].

Recent developments in precision nanomedicine, stimuli-responsive nanoplatforms, theranostic systems, and advanced targeting strategies are expected to improve the clinical applicability of neuroprotective nanomaterials [73]. In parallel, the adoption of human-derived organoids, organ-on-chip technologies, and standardized regulatory frameworks may facilitate the generation of more predictive preclinical data and accelerate translational progress.

Overall, current evidence suggests that the future success of neuroprotective nanomedicine will depend not only on the development of increasingly sophisticated nanomaterials but also on the establishment of robust translational pipelines capable of bridging the gap between experimental neuroprotection and clinical neurorepair. The integration of safety assessment, scalable manufacturing, regulatory harmonization, and patient-specific therapeutic design will be essential for the successful implementation of next-generation nanotherapeutics in neurological disorders [75,76].

## 6. Conclusions and Future Perspectives

Neuroprotective nanomaterials are emerging as a transformative approach in the management of CNS injury, offering the unique ability to simultaneously modulate multiple interconnected pathological mechanisms. As highlighted throughout this review, CNS injury is characterized by a complex interplay of molecular and cellular processes that cannot be effectively addressed by single-target therapies. Consequently, advanced nanomaterials may offer an integrated strategy to target oxidative stress, neuroinflammation, mitochondrial dysfunction, and impaired regeneration. This multifunctional behavior represents a critical step toward bridging the gap between neuroprotection and neurorepair. However, despite their considerable promise, several challenges continue to limit clinical translation, including safety concerns, targeting limitations, manufacturing complexity, regulatory uncertainty, and the need for more predictive preclinical models. Addressing these issues will be essential to fully unlock the therapeutic potential of nanotechnology in neurological disorders.

Future developments are likely to focus on intelligent and multifunctional nanoplatforms capable of dynamically responding to the pathological microenvironment and integrating neuroprotective and regenerative functions within a single therapeutic system. In this context, emerging approaches such as stimuli-responsive nanomaterials may improve therapeutic precision by enabling site-specific and disease-triggered drug release within the injured CNS, while nanozymes have attracted increasing interest due to their ability to mimic endogenous antioxidant enzymes and simultaneously modulate oxidative stress and neuroinflammation. In parallel, advances in stem cell engineering, gene therapy, RNA-based therapeutics, and personalized medicine are expected to further expand the scope of nanotechnology-based interventions. Importantly, the evidence discussed in this review supports a conceptual transition from a material-centered perspective of nanomedicine toward a function-oriented framework that prioritizes biological activity and therapeutic outcome. This perspective may facilitate the rational development of next-generation neuroprotective strategies by aligning nanomaterial design with specific therapeutic objectives.

The function-based classification proposed in this review provides a conceptual framework for the rational design of next-generation neuroprotective nanotherapeutics, helping to align material development with biologically relevant mechanisms of neuroprotection, neuroregeneration, and functional recovery across diverse CNS disorders.

## Figures and Tables

**Table 1:** Conventional Material-Based Classification of Neuroprotective Nanomaterials.

Nanomaterial Class	Representative Materials	Main Physicochemical Features	Principal Advantages in CNS Applications	Major Limitations	References
Carbon-based nanomaterials	Graphene, graphene oxide (GO), carbon nanotubes (CNTs)	High surface area, electrical conductivity, mechanical strength	Neural interfacing, promotion of neuronal differentiation, support of synaptic activity and neurogenesis	Potential toxicity related to dose, size, and surface functionalization	[25]
Polymeric nanoparticles	PLGA, chitosan, PEG-based systems	Biodegradability, biocompatibility, tunable release kinetics	Controlled drug delivery, enhanced bioavailability, BBB targeting through surface modification	Limited intrinsic neuroprotective activity and potential release variability	[14]
Lipid-based nanocarriers	Liposomes, solid lipid nanoparticles (SLNs), nanostructured lipid carriers (NLCs)	Membrane-like structure, high biocompatibility	Efficient encapsulation of therapeutic compounds, improved BBB penetration	Stability issues and limited drug-loading capacity	[4]
Inorganic nanomaterials	Gold nanoparticles, silica nanoparticles, cerium oxide nanoparticles	High physicochemical stability, tunable size and shape	Antioxidant activity, anti-inflammatory effects, theranostic applications	Potential bioaccumulation and long-term safety concerns	[26]
Hybrid nanomaterials	Polymer-lipid hybrids, graphene-polymer composites, organic-inorganic systems	Multifunctionality, combined physicochemical properties	Simultaneous targeting of multiple pathological pathways	Complex manufacturing and scalability challenges	[27]
Stimuli-responsive nanomaterials	Redox-sensitive, pH-sensitive and enzyme-responsive nanoparticles	Environment-responsive activation	Site-specific delivery and controlled therapeutic release	Limited clinical validation	[23]
Nanozymes	Cerium oxide, iron oxide, manganese oxide and Prussian blue nanoparticles	Enzyme-mimicking catalytic activity	Potent ROS scavenging and modulation of inflammatory responses	Need for extensive long-term safety assessment	[24]

**Table 2:** Function-Based Classification of Neuroprotective Nanomaterials and Their Contribution to Functional Neurorepair.

Functional Category	Representative Nanomaterials	Primary Biological Targets	Main Neuroprotective Effects	Contribution to Neurorepair	References
Antioxidant nanomaterials	Nanoceria, nanozymes, graphene derivatives	ROS, mitochondrial dysfunction, oxidative stress pathways	Reduction in oxidative damage and preservation of mitochondrial homeostasis	Indirect support of regeneration through limitation of secondary injury	[28]
Anti-inflammatory nanomaterials	Gold nanoparticles, polymeric nanoparticles, lipid nanocarriers	Microglia, astrocytes, NF-κB signaling, inflammatory cytokines	Attenuation of neuroinflammation and modulation of glial activation	Promotion of a permissive regenerative microenvironment	[6]
Anti-apoptotic nanomaterials	Polymeric nanoparticles, hybrid nanoplatforms, mitochondria-targeted nanosystems	Caspases, cytochrome c release, PI3K/Akt and MAPK pathways	Increased neuronal survival and resistance to injury	Preservation of neuronal populations available for repair	[8]
Neurogenic nanomaterials	Graphene-based materials, conductive biomaterials, stem-cell instructive nanoscaffolds	Neural stem cells and progenitor cells	Enhanced stem cell proliferation and neuronal differentiation	Potential contribution to neuronal replacement through enhanced neurogenesis	[9]
Synaptogenic and neuroplastic nanomaterials	Graphene, carbon nanotubes, conductive hybrid systems	Synaptic proteins, neuronal excitability, network activity	Enhancement of synaptic transmission and plasticity	Potential support for restoration of neural connectivity	[13]
Regenerative nanomaterials	Electrospun nanofibers, hydrogel-based scaffolds, nerve guidance conduits	Axonal growth, extracellular matrix remodeling, cell adhesion pathways	Structural support for tissue repair and axonal guidance	Promotion of axonal regeneration and structural repair	[29]
Multifunctional nanomaterials	Hybrid nanoparticles and multifunctional nanoplatforms	Multiple pathological pathways simultaneously	Combined antioxidant, anti-inflammatory and anti-apoptotic activities	Integrated neuroprotection and neurorepair	[21]

**Table 3:** Translational and Experimental Evidence Supporting Functional Neuroprotective Nanomaterials.

Functional Category	Principal Mechanism	Representative Nanomaterials	In Vitro Evidence	In Vivo Evidence	Translational Relevance	Level of Supporting Evidence	References
Antioxidant	Reduction in ROS production, free radical scavenging, restoration of redox homeostasis	Cerium oxide nanoparticles, selenium nanoparticles, fullerene derivatives	Protection against oxidative stress-induced neuronal damage; improved cell viability	Reduced oxidative injury and neuronal loss in models of stroke, SCI, and neurodegeneration	Particularly relevant for oxidative stress-driven neurological disorders	Strong molecular and cellular evidence; substantial support from small-animal models; limited clinical evidence	[28]
Anti-inflammatory	Modulation of microglial activation, suppression of pro-inflammatory cytokines, regulation of neuroimmune responses	Gold nanoparticles, curcumin-loaded nanoparticles, PLGA-based systems	Reduced expression of TNF-α, IL-1β, and other inflammatory mediators	Attenuation of neuroinflammation and secondary tissue damage	Supports control of chronic and acute neuroinflammatory processes	Strong preclinical evidence, predominantly derived from rodent models; limited human data	[6]
Anti-apoptotic	Inhibition of apoptotic signaling pathways, modulation of Bcl-2/Bax balance, reduction in caspase activation	Neurotrophic factor-loaded nanoparticles, peptide-functionalized nanocarriers	Reduced neuronal apoptosis and enhanced cell survival	Preservation of neuronal populations after CNS injury	Particularly valuable during acute and subacute phases following injury	Mechanistic evidence supported by cellular studies and experimental CNS models; limited functional validation	[8]
Neurogenic	Promotion of neural stem/progenitor cell proliferation and differentiation	Chitosan nanoparticles, growth factor-loaded nanocarriers, self-assembling nanomaterials	Enhanced neuronal differentiation and neurite formation	Increased neurogenesis and endogenous repair responses	Facilitates regeneration through stimulation of resident progenitor cells	Evidence primarily based on cellular, molecular, and histological outcomes	[9]
Synaptogenic/Neuroplastic	Enhancement of synaptic remodeling, neurite extension, and network reorganization	Graphene-based nanomaterials, conductive nanomaterials, neurotrophic factor-delivering nanoparticles	Increased synaptic marker expression and neurite connectivity	Improved functional recovery associated with neural circuit remodeling	Supports restoration of neuronal connectivity and functional plasticity	Moderate preclinical evidence; electrophysiological and functional validation remains comparatively limited	[13]
Regenerative	Promotion of axonal regeneration, tissue reconstruction, and structural repair	Nanofiber scaffolds, hydrogel-based nanocomposites, extracellular vesicle-inspired nanomaterials	Improved cell adhesion, migration, and axonal growth	Enhanced tissue repair and axonal regeneration in CNS and PNS models	Supports long-term structural recovery after injury	Strong structural and histological evidence in animal models; functional recovery outcomes remain variable	[29]
Multifunctional	Simultaneous modulation of multiple neuroprotective pathways	Hybrid nanosystems, multifunctional polymeric platforms, biomimetic nanocarriers	Combined antioxidant, anti-inflammatory, neurogenic, and regenerative effects	Superior therapeutic outcomes through synergistic mechanisms	Represents the most promising strategy for complex neurological disorders	Broad preclinical support combining multiple endpoints; clinical translation remains at an early stage	[21]

**Table 4:** Critical appraisal of current applications of neuroprotective nanomaterials in neurological disorders.

Clinical Setting	Main Therapeutic Targets	Principal Advantages of Nanomaterials	Major Limitations	Current Level of Evidence	Translational Readiness	References
Neurodegenerative diseases (AD, PD)	Oxidative stress, protein aggregation, neuroinflammation, mitochondrial dysfunction	Multitarget modulation of disease mechanisms; improved CNS delivery; potential neurogenic activity	Limited predictive value of animal models; lack of long-term efficacy data; disease heterogeneity	Predominantly preclinical	Low–Moderate	[52,53]
Ischemic stroke	Oxidative stress, inflammation, BBB disruption, neuronal death	Simultaneous neuroprotective and regenerative effects; promotion of angiogenesis and neurogenesis	Narrow therapeutic window; difficulty reproducing clinical heterogeneity; limited human data	Strong preclinical evidence	Moderate	[54,56]
Traumatic brain injury	Secondary injury cascade, neuroinflammation, ROS production, mitochondrial dysfunction	Ability to target prolonged injury processes; antioxidant and anti-inflammatory activity; regenerative support	Lack of standardized models; limited clinical translation; safety concerns	Preclinical	Moderate	[57,58]
Peripheral nerve injury	Axonal degeneration, inflammation, impaired nerve regeneration	Excellent platform for evaluating neurorepair; effective scaffold integration; direct regenerative activity	Biological differences between PNS and CNS may limit extrapolation	Robust preclinical evidence	Moderate–High	[9,59,60]

## Data Availability

No new data were created or analyzed in this study. Data sharing is not applicable to this article.

## Abbreviations

The following abbreviations are used in this manuscript:

CNS	Central Nervous System
TBI	Traumatic Brain Injury
BBB	Blood–Brain Barrier
BSCB	Blood-Spinal Cord Barrier
GO	Graphene Oxide
CNTs	Carbon Nanotubes
PLGA	Poly-Lactic-co-Glycolic Acid
SLNs	Solid Lipid Nanoparticles
NLCs	Nanostructured Lipid Carriers
ROS	Reactive Oxygen Species
ATP	Adenosine Triphosphate
AD	Alzheimer’s Disease
PD	Parkinson’s Disease
PNI	Peripheral Nerve Injury
